# Natural and anthropogenic carbon input affect microbial activity in salt marsh sediment

**DOI:** 10.3389/fmicb.2023.1235906

**Published:** 2023-09-07

**Authors:** Erin S. Frates, Rachel L. Spietz, Michael R. Silverstein, Peter Girguis, Roland Hatzenpichler, Jeffrey J. Marlow

**Affiliations:** ^1^Department of Biology, Boston University, Boston, MA, United States; ^2^Department of Chemistry and Biochemistry, Montana State University, Bozeman, MT, United States; ^3^Center for Biofilm Engineering, Montana State University, Bozeman, MT, United States; ^4^Bioinformatics Program, Boston University, Boston, MA, United States; ^5^Department of Organismic and Evolutionary Biology, Harvard University, Cambridge, MA, United States; ^6^Department of Microbiology and Cell Biology, Montana State University, Bozeman, MT, United States; ^7^Thermal Biology Institute, Montana State University, Bozeman, MT, United States

**Keywords:** microbial diversity, metabolic activity, salt marsh, carbon cycle, biogeochemistry

## Abstract

Salt marshes are dynamic, highly productive ecosystems positioned at the interface between terrestrial and marine systems. They are exposed to large quantities of both natural and anthropogenic carbon input, and their diverse sediment-hosted microbial communities play key roles in carbon cycling and remineralization. To better understand the effects of natural and anthropogenic carbon on sediment microbial ecology, several sediment cores were collected from Little Sippewissett Salt Marsh (LSSM) on Cape Cod, MA, USA and incubated with either *Spartina alterniflora* cordgrass or diesel fuel. Resulting shifts in microbial diversity and activity were assessed via bioorthogonal non-canonical amino acid tagging (BONCAT) combined with fluorescence-activated cell sorting (FACS) and 16S rRNA gene amplicon sequencing. Both *Spartina* and diesel amendments resulted in initial decreases of microbial diversity as well as clear, community-wide shifts in metabolic activity. Multi-stage degradative frameworks shaped by fermentation were inferred based on anabolically active lineages. In particular, the metabolically versatile *Marinifilaceae* were prominent under both treatments, as were the sulfate-reducing *Desulfovibrionaceae*, which may be attributable to their ability to utilize diverse forms of carbon under nutrient limited conditions. By identifying lineages most directly involved in the early stages of carbon processing, we offer potential targets for indicator species to assess ecosystem health and highlight key players for selective promotion of bioremediation or carbon sequestration pathways.

## 1. Introduction

Coastal salt marsh sediments are highly productive ecosystems exhibiting diverse metabolic functions that vary over both space and time. Abundant organic carbon input leads to anoxic conditions near the sediment-water interface, and seawater sulfate concentrations drive carbon cycling in deeper horizons to generate reducing conditions that support chemosynthetic metabolisms. The result is a compressed set of distinct redox zones that host high levels of microbial biodiversity (Lozupone and Knight, 2007; Armitage et al., 2012) and promote metabolic strategies that span reduced and oxidized microenvironments (Seitz et al., 1993; Larsen et al., 2015).

Organic carbon provides the anabolic and catabolic fuel for marsh-hosted microbial activity. Understanding the source, fate, and community-wide repercussions of distinct carbon inputs represents an important priority for estimating blue carbon budgets. The dominant source of organic carbon in eastern North American salt marshes is the cordgrass *Spartina alterniflora*, which builds stems and leaves up to 1.5 meters above the water’s surface as well as a dense root network below ground (Darby and Turner, 2008). At the Great Sippewissett salt marsh on Cape Cod, Massachusetts, *S. alterniflora* accounted for 212 g cm^–2^ year^–1^ of net primary productivity–four times more than the contribution from microalgal species (Van Raalte et al., 1976). When the grass’s above-ground lignocellulose biomass is shed, ∼80% of it falls to the water-sediment interface in salt marsh pools and is decomposed *in situ*, with remineralization of the cellulose moiety outpacing that of the lignin (Hodson et al., 1984). In an early study at Great Sippewissett, the degradation process reached its peak rate after just 4 days and continued for more than 2 years thereafter (Wilson, 1985). Unlike in terrestrial settings, where fungi play a primary role in lignocellulose breakdown (Andlar et al., 2018), decomposition in salt marsh sediments is mediated by fungi, bacteria, and archaea (Benner et al., 1984). When ^13^C-labeled lignocellulose was introduced to a 30-day, lab-based incubation of the top centimeter of salt marsh sediment from Huntington Beach, CA, *Desulfosarcina*, *Spirochetes*, *Kangiella*, and *Sediminibacter* were most over-represented in the isotopically enriched community (Darjany et al., 2014). A metaproteomics study in the Welwick, UK, salt marsh identified 42 bacterial families that were excreting glycoside hydrolases to break down lignocellulose at the sediment-water interface (Leadbeater et al., 2021).

The ways in which salt marsh microbial communities are affected by anthropogenic factors remain poorly understood. Given their position at the terrestrial-marine interface, salt marshes face pollutants from both sides: runoff and fluvial input from inland sources (Valiela and Cole, 2002), and wave-deposited pollutants (e.g., oil spill products) from marine and submarine settings (Vega et al., 2009; Natter et al., 2012). The natural repository of diverse organisms and the presence of distinct redox zones position salt marsh sediments well for the stepwise detoxification and decomposition of toxic organic molecules (Reddy and DeLaune, 2008), though recalcitrant components can persist for decades (Reddy et al., 2002). It has been suggested that microbial communities in salt marshes can consume diesel fuel and indeed have adapted to do so in frequently-impacted areas (Carman et al., 1996). A time series analysis of Louisiana salt marsh sediments impacted by the Deepwater Horizon oil spill revealed anabolic processing of petroleum products, concomitant with increased proportions of *Rhodobacterales* and *Sphingomonadales* (Mahmoudi et al., 2013).

Nonetheless, the lack of spatial, temporal, and phylogenetic resolution pertaining to organic pollutant degradation precludes a mechanistic understanding that would help track persistent exposure and facilitate the design of microbial communities for bioremediation purposes. Considering the importance of salt marshes as “blue carbon sinks” (Duarte et al., 2010; Mcleod et al., 2011) whose long-term stability and ecosystem function are threatened by rising sea levels (FitzGerald and Hughes, 2019) and increased exposure to organic pollutants (Girones et al., 2021), deeper knowledge of carbon cycle dynamics in salt marsh sediments is urgently needed.

In this study, we evaluated the effect of organic carbon loading–both natural and anthropogenic–on microbial community structure. In particular, we aimed to identify the microbial community members that are preferentially anabolically active upon surficial deposition of (a) *S. alterniflora* biomass, or (b) diesel fuel, as well as the broader community effects of natural and anthropogenic carbon loading.

Linking microbial identity with metabolic function remains a challenge in environmental systems. Here, we used substrate analog probing (Hatzenpichler et al., 2020) through bioorthogonal non-canonical amino acid tagging (BONCAT) to fluorescently tag and, via fluorescence-activated cell sorting (FACS) and high-throughput gene amplicon sequencing, subsequently identify anabolically active microbes. BONCAT appears to be a taxonomically agnostic approach with no measurable effect on community composition in environmental microbiomes (reviewed in Hatzenpichler et al., 2020) and imposes only a minor influence on protein synthesis and metabolome in *E. coli* cultures (Bagert et al., 2014; Hatzenpichler et al., 2014; Steward et al., 2020); however, detrimental effects on the growth dynamics of *Synechococcus* sp. have been reported (Michels et al., 2021). BONCAT has been an effective tool for ecophysiological studies in ocean water (Samo et al., 2014; Leizeaga et al., 2017; Sebastián et al., 2019), marine sediments (Hatzenpichler et al., 2016; Krukenberg et al., 2021), geothermal habitats (Marlow et al., 2020; Reichart et al., 2020), soils (Couradeau et al., 2019), salt marsh sediments (Marlow et al., 2021), the terrestrial subsurface (McKay et al., 2022; Schweitzer et al., 2022), waste water (Du and Behrens, 2021; Madill et al., 2021), and host-associated microbiomes (Riva et al., 2020; Valentini et al., 2020; Taguer et al., 2021a,b).

At Little Sippewissett Salt Marsh (LSSM) in Falmouth, MA, a range of BONCAT experiments was conducted: sediment was incubated *in situ* in customized chambers for up to 3 weeks in the presence of *S. alterniflora* or diesel fuel to identify the microbial constituents that were responsive to natural and anthropogenic carbon, respectively. A total of 16S rRNA gene surveys of both the anabolically active and inactive communities were performed at discrete sediment horizons to track the effects of surficial carbon on deeper communities. Our findings demonstrate that carbon amendments first narrow and then expand microbial diversity among anabolically active organisms, and that substantial shifts in the composition of anabolically active communities can be resolved within days of carbon amendment. Some taxa respond preferentially to either *Spartina* or diesel addition, while the response of others seems to be more agnostic regarding the source of carbon. By gaining enhanced temporal and spatial resolution on how carbon flows through the LSSM sediment ecosystem, this work illuminates the roles of specific microbial taxa in carbon and nutrient cycling in salt marsh ecosystems.

## 2. Materials and methods

### 2.1. Study site description and incubation conditions

**FIGURE 1:**
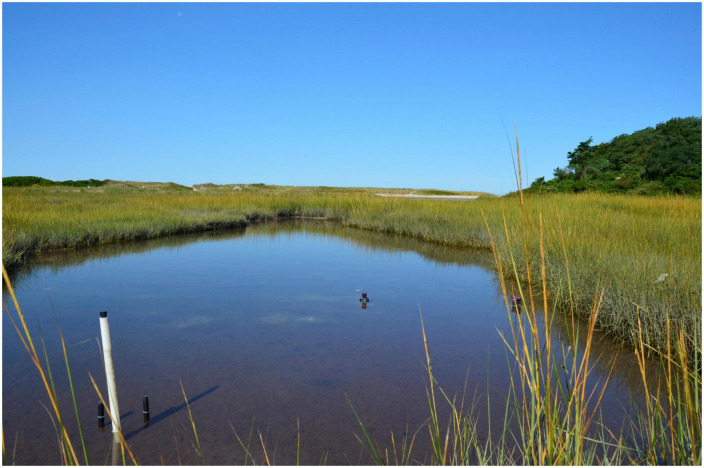
Photograph of *in situ* core incubations in the Berry Pond at Little Sippewissett Salt Marsh.

*In situ* incubations were conducted in the “Berry Pool” located at 41.5758° latitude, -70.6394° longitude within Little Sippewissett Salt Marsh (LSSM; Figure 1) on Cape Cod, MA, USA in June (carbon experiment) and August (replication experiment) of 2019. LSSM sediments are marked by dramatic redox gradients; previous studies reported anoxic porewaters below 5 mm depth, sulfide rising from 0 to 1.5 mM in the top cm, and pH falling 1–2 units immediately below the surface, stabilizing around 7.0–7.3 at night and 6.0 during the day (Larsen et al., 2015; Salman et al., 2015). The Berry Pool is surrounded by *Spartina* grass, whose degradation products frequently fall into the pool; despite the regional impacts of a 1,969 oil spill (Reddy et al., 2002), LSSM has not been reported to exhibit any lasting effects of hydrocarbon pollution (Little Sippewissett Marsh, n.d.).

Custom core sleeves were constructed using glycol-modified polyethylene terephthalate (PETG) tubing (2.54 cm outer diameter, 1.91 cm inner diameter, McMaster-Carr, Elmhurst, IL, USA) cut to ∼20 cm length. PETG was selected because of its low gas permeability, high optical transparency, and high rigidity for downstream transport and embedding (Fisher Scientific, Waltham, MA, USA). These properties minimized the exposure of subsurface horizons to oxygen during transport but allowed sunlight to reach surficial layers during the incubation period. Cut-off threaded ends of conical centrifuge tubes were attached to the ends of the core sleeves using Master Plumber epoxy putty (William H. Harvey, Omaha, NE, USA) to allow caps to be screwed on to minimize gas exchange and fluid leakage during transport to and from the field site (Additional details on the customized cores can be found in Marlow et al., 2021).

To begin the experimental process, sediment cores consisting of ∼10–12 cm of sediment and ∼8–10 cm of overlying water were collected, and the bottom of each core sleeve was packed with autoclave-sterilized glass wool to prevent sediment loss. Caps were screwed on, and the filled core sleeves were placed in a dark cooler, transported to the lab (∼20 min away), and moved into an anoxic chamber (3.5% H_2_, 20% CO_2_, 76.5% N_2_) to reduce oxygen exposure. All cores were gravity-flushed with three core volumes of 0.22 μm filter sterilized seawater, which was collected from the Berry Pool at the same time as core recovery. This method of fluid replacement was selected to minimize physical disruptions, exerting pressure differentials no greater than those associated with naturally occurring tidal fluctuations.

Experimental cores were prepared as shown in Table 1. L-Homopropargylglycine (HPG, Click Chemistry Tools, Scottsdale, AZ, USA), a non-canonical amino acid often used for BONCAT experiments, was added directly to filter-sterilized seawater (reaching a final concentration of 50 μM) and introduced during the three column-volume flushes for treatments B, C, D, F, and G (Table 1). Once the fluids were in place, the bottom cap was screwed on, with a 0.25 mm-thick silicone polydimethylsiloxane (PDMS) membrane (Interstate Specialty Products, Sutton, MA) secured between the cap and the tube. PDMS is gas-permeable and liquid-impermeable, ensuring the incubation volume retained the HPG and carbon amendments while remaining in contact with the environment’s gaseous chemical species.

**TABLE 1:** Details on the treatment conditions for the *in situ* incubations conducted in Little Sippewissett Salt Marsh.

Experiment	ID and treatment	HPG	Diesel	*Spartina*	Incubation duration
Natural organic amendment	A: Control	0 μM		No	21 days
	B: HPG control	50 μM		No	21 days
	C: *Spartina* amendment	50 μM		Yes	21 days
	D: *Spartina* amendment	50 μM*		Yes	21 days
Anthropogenic organic amendment	E: Control	0 μM	No		4 days
	F: HPG control	50 μM	No		4 days
	G: Diesel amendment	50 μM	Yes		4 days

*HPG was added during the final 4 days of incubation.

For treatments C and D, 1 g per core of *S. alterniflora* above-ground biomass (collected during core collection and transported in sterile containers), was cut into ∼1.5-cm long pieces and gently placed on the top of the sediment. For treatment G, 500 mM of number two diesel fuel (referred to throughout as “diesel”) was pipetted into the overlying water column, which was gently mixed to result in a final concentration of 25 mM (The calculation of overlying water volume was based on the core’s inner diameter and column height).

After all amendments were added, the cores were sealed with PDMS and twist-on caps and transported in the dark back to the Berry Pool in LSSM after approximately 4 h of processing in the lab. Prior to placing the cores back into the sediment, the end caps (but not the PDMS membranes) were punctured several times with a pin in order to allow for gas diffusion to/from the cores. Cores remained in the Berry Pool until collection following either 4 days or 21 days, depending on the designated incubation time (Table 1). These incubation durations were used in order to detect organisms across multiple diel cycles, and to ensure that the more metabolically challenging lignocellulose (i.e., *Spartina*) had sufficient time to be degraded. One core (treatment D) received *S. alterniflora* at the beginning of the three-week incubation and was then removed from LSSM after 17 days in order to add HPG (through fluid replacement as described above) for the last 4 days of the incubation. This delayed HPG-addition was used to capture a snapshot of microbial community composition and activity during later-stage *Spartina* degradation.

### 2.2. Sample preservation

At the end of each experiment’s incubation period, the cores were removed from LSSM, and electrical tape was used to seal the bottom pinholes to minimize oxygen infiltration to the core’s anoxic horizons during transport to the lab. The cores were placed in a dark cooler and taken to the lab’s anoxic chamber for processing. All cores were infiltrated with 3% paraformaldehyde (PFA) in filtered seawater (using three column volumes to ensure full infiltration) and incubated at room temperature for 1 h to cease metabolic activity and fix all cells. The PFA was then washed from the core with three column volumes of 0.22 μm-filtered seawater.

To constrain the effect of experimental treatments on sequence data, a five-day incubation with HPG following a 1-h fixation with 2% PFA generated < 0.05% of BONCAT+ cells compared with an unfixed control (a proportion similar to the false positive rate in an HPG-free incubation; data not shown). This validation offers confidence that our PFA treatment ceased HPG incorporation, and that the BONCAT+ signal reflected *in situ* anabolic activity.

The cores were sealed with sterilized glass wool atop 2–3 cm of filtered seawater overlying the sediment surface to minimize sample disruption during shipment to Montana State University. Additional filtered seawater was added above the glass wool, and the core was capped with new conical tube caps. Cores were shipped overnight on ice, where they were kept at 4°C until further processing. Minimal sediment disruption was detected during transport.

### 2.3. Cell extraction and click reaction

Sediment cores were carefully excised from the core sleeve using a custom-built, sterilized plunger. Cores were divided into 1 cm increments, labeled as follows: L1 (0–1 cm depth horizon), L2 (1–2 cm), L3 (2–3 cm), L4 (3–4 cm), and L5 (4–5 cm). A total of 0.5 g of each horizon was preserved for bulk DNA analysis, and the remaining sediment was weighed, transferred to a 50 mL conical tube containing 10 mL of sterilized 1x PBS, and stored at 4°C until cells were extracted. From each core, the top 5 layers were processed to focus on the oxic-anoxic interface and the sediment horizons most influenced by surficial additions.

Cells were extracted from each sediment layer following methods used by Couradeau et al. (2019) with modifications. For each horizon, 1 mL of sediment slurry was diluted with 5 mL of sterile 1 × PBS in a 15 mL conical tube with 0.02% Tween 20 (MP Biomedicals, Santa Ana, CA, USA). This solution was vortexed at maximum speed for 5 min. Large sediment particles were pelleted via centrifugation at 500 × *g* for 55 min. Cells from 700 μL of the supernatant were pelleted in a 1.5 ml microcentrifuge tube by centrifugation at 16,000 × *g* for 5 min. The supernatant was removed before the click reaction (detailed below) was performed directly on the cell pellet. Extraction blanks were performed without any added sediment in parallel with cell extractions to test for reagent contamination.

The click reaction solution was prepared in a large volume in order to stain all samples using the same solution. The reaction solution contained the following components: 5 mM amino guanidine hydrochloride (Sigma Aldrich, St. Louis, MO, USA), 5 mM sodium *L*-ascorbate (Sigma Aldrich, St. Louis, MO, USA), 100 μM copper sulfate pentahydrate (Sigma Aldrich, St. Louis, MO, USA), 500 μM THPTA (Click Chemistry Tools), 4 μM Cy5 picolyl-azide dye (Click Chemistry Tools), and 0.25 × SYBR Green I (ThermoFisher, Waltham, MA, USA) in 1 × PBS. A total of 250 μL of the click reaction solution was applied to each cell pellet, which was resuspended via gentle pipetting. The samples with reaction solution were rotated in the dark at room temperature for 1 hr. Cells were then washed by three rounds of (1) centrifugation at 16,000 × *g*, (2) supernatant removal, and (3) resuspension in 1 mL of 1 × PBS. Finally, click-stained cells were resuspended in 500 μL of 1 × PBS, passed through a 35 μm filter cap (Electron Microscopy Sciences, Hatfield, PA, USA), and stored in the dark at 4°C until sorting. Cells were extracted from all samples, click stained, and sorted within a 24-h window.

### 2.4. Cell sorting

Cells were sorted based on the presence of SYBR Green I fluorescence and BONCAT signal (Cy5 fluorescence) intensity using a Sony SH800S FACS. For sorting, a first gate was drawn based on forward scatter area (FSC-A) and back scatter area (BSC-A) to exclude any large sediment particles that remained after filtration. A second gate was then drawn based on forward scatter width (FSC-W) and forward scatter height (FSC-H) to further constrain the size of particles examined. Three cell fractions from each sample were sorted based on FSC-A and fluorescence intensity: all cells (SYBR green I fluorescence), active cells (SYBR green I fluorescence and Cy5 fluorescence), and inactive cells (SYBR green I fluorescence and no Cy5 fluorescence). The “all cells” fraction was gated on FSC-A, and SYBR Green I fluorescence of SYBR stained cells and was determined by comparison to a non-SYBR stained sample control. All events that were identified as cells on the SYBR Green I gate were further gated into active (BONCAT positive) or inactive (BONCAT negative) fractions based on Cy5 fluorescence intensity. A no-HPG sample was used as the negative control to draw the BONCAT positive gate on the Cy5 channel. As in Couradeau et al. (2019), a sterile 96-well plate was preloaded with 25 μL of sterile milliQ water in each well. Cells for each fraction of each sample were sorted into a 96-well plate in duplicate. For the “all cells” fraction, 100,000 events were sorted for each fraction in duplicate. Sterile sheath fluid was collected from the cell sorter for use as processing controls to identify potential contaminants during the sorting and process.

### 2.5. DNA extraction and 16S rRNA gene sequencing

Once sorting was completed, the 96-well sample plates were centrifuged at 4,700 × *g* for 95 min at 10°C, and 100 μL of the overlying supernatant was removed by pipette. A second, sterile 96-well plate was taped atop the plate containing sorted cells, then inverted to catch the remaining supernatant during a brief centrifugation step at 45 × *g* for 10 seconds. The 96-well plate containing the supernatant was carefully removed and discarded, and remaining cell pellets were resuspended in 20 μL of sterile, nuclease free water. To lyse sorted cells and extract DNA, the 96-well plates were subjected to three rounds of freeze/thaw at −80°C for 20 min and 99°C for 10 min.

Following methods used in successful BONCAT-FACS experiments by Reichart et al. (2020) and Krukenberg et al. (2021), bacterial and archaeal 16S rRNA genes were amplified by 36 cycles of PCR (28 cycles for rRNA gene amplification followed by 8 cycles for barcoding) using the Earth Microbiome protocol with updated 515F (5′-GTGYCAGCMGCCGCGGTAA-3′) and 806R (5′-GGACTACNVGGGTWTCTAAT-3′) primers directly in the 96-well plates containing extracted DNA from sorted cells. Briefly, into each well containing 20 μL of cell lysate, 20 μL of Invitrogen Platinum Taq II 2X Master Mix, 1 μL of each primer (final concentration 0.2 μM each), and 5 μL of nuclease free water were added. Successful amplification was verified by gel electrophoresis. The PCR products were purified using AMPure XP beads (Beckman Coulter, Brea, CA, USA) cleanup following the manufacturer’s protocol with a final elution in 40 μL of nuclease free water. A second eight-cycle PCR was used to attach barcodes and sequencing adapters to the purified 16S rRNA gene amplicons generated in the first PCR. The second PCR contained 12 μL Invitrogen Platinum Taq II 2X Master Mix, 2.5 μL i5 primer (final: 0.25 μM), 2.5 μL i7 primer (final: 0.25 μM), and 2.5 μL water. The final products were purified again using AMPure XP beads and were quantified using the Quant-iT Picogreen dsDNA Assay (Invitrogen, Waltham, MA, USA). Purified, barcoded PCR products were pooled at equimolar concentration of 10 ng DNA each and sequenced using Illumina MiSeq 2 × 250 bp technology at Idaho State University’s Molecular Research Core Facility (Pocatello, ID, USA). Sequences were processed as previously described (Marlow et al., 2021) to demultiplex, remove primers, trim, filter, denoise, merge paired reads, remove chimeras, determine amplicon sequence variants (ASVs) with *DADA2* (Callahan et al., 2016), assign taxonomy with the SILVA 132 database, filter contaminant sequences, and normalize read count across samples. Sequences have been archived at NCBI under the Bioproject ID PRJNA973662.

### 2.6. Community analysis

In order to compare species richness and abundance of the microbial communities associated with each sample, we calculated Shannon, Simpson, and Chao alpha diversity metrics through the vegan (V2.5-7) package run and phyloseq (V1.36.0) package in Rstudio (4.1.1; Posit Team, 2022; R Core Team, 2022) using rarified ASV abundance data. The Chao value, s_*p*_, quantifies overall species richness in a sample and is sensitive to the number of singleton representatives. Higher values reflect more ASVs and a higher number of singletons. It is calculated according to equation 1:


(1) $$s_{p}=s_{o}+\frac{a_{1}^{2}}{2\,a_{2}}\cdot \frac{\left(N-1\right)}{N}$$

where s_*o*_ is the number of ASVs, a_1_ refers to the number of singletons, or ASVs occurring only once, and a_2_ refers to doublets, or ASVs occurring twice. N is the number of sequences included in the analysis.

The Simpson diversity metric (Eq. 2) measures the diversity within a community, taking into account both the richness and evenness. Higher values indicate a more consistent distribution of species (assuming a constant number of ASVs).


(2) $$1-\left[\Sigma \,p_{i}^{2}\right]$$

The Shannon diversity metric (*H*, Eq. 3) is particularly sensitive to species that account for a smaller proportion of the population. Higher values indicate a more even distribution of species, whereas a lower value reflects a greater number of ASVs (In Eqs. 2 and 3, *p*_*i*_ is the relative abundance of a given species in the community).


(3) $$H=-\Sigma \,p_{i}\,l\,n\,\left(p_{i}\right)$$

Aitchison distances were calculated in order to assess the beta diversity between samples while accounting for the compositional artifacts that sequencing data is prone to Gloor et al. (2017). This distance measure is determined by taking the Euclidean distance of center-log transformed ASV data, which is used to normalize samples according to read count. The center-log transformation was performed using the compositions (V2.0-4) package in RStudio (V4.1.1), as shown in Eq. 4, where x is a matrix of compositional data.


(4) $$c\,l\,r\,\left(x\right)=\left(l\,n\,x_{i}-\frac{1}{D}\,\sum_{j=1}^{D}l\,n\,x_{j}\right)$$

A distance matrix was then generated with the subsequent count data using the rdist (V0.0.5) package in RStudio (V4.1.1), as shown in Eq. 5, where v and w each refer to a given vector.


(5) $$\sqrt{\Sigma_{i}\,{\left(v_{i}-w_{i}\right)}^{2}}$$

Results reflect the magnitude by which communities differ; the greater the distance between two communities, the more dissimilar their compositions. Once determined, the Aitchison distances between samples were visualized with multiple dimensional scaling (MDS) using the phyloseq (V1.36.0) package and visualized with ggplot2 (V3.3.5) in RStudio (V4.1.1). The multivariate normal distribution of each data subset was depicted with ellipses. Finally, community level differences were tested for statistical significance using PERMANOVA tests.

The relative abundance of taxa at each horizon in each core was determined for a range of secondary analyses. Taxa accounting for less than 5% relative abundance were pooled into a subset entitled “Low-Abundance Taxa.” Phylum-level results were visualized in ggplot2 (V3.3.5); low-abundance taxa were aggregated as “Other.” Family-level results were used to calculate the ratio of active:inactive cells within like-treatments and active:active cells between control and treated cores. Since controls A and E lacked HPG, cellular activity could not be determined; therefore, ASV counts from the active and inactive fractions in the remaining treatments were pooled *in silico* in order to construct communities representative of the whole community, regardless of anabolic activity status. These pools were then rarefied to account for superficially high reads. The resulting communities were compared to groups A and E to determine whether the presence of HPG affected community structure within the BONCAT cores.

### 2.7. Method validation

To determine whether HPG addition influences microbial community composition, sequence data from two pairs of treatments (E vs. F and A vs. B) were compared. Pooled communities from treatments F and B were used to provide the most suitable comparison with HPG-free treatments E and A, which could not be divided into active and inactive subsets. To determine how *in situ* incubation time influenced the structure of microbial communities with active/inactive resolution, we compared control treatment B (a 21-day field incubation) with control treatment F (a four-day field incubation). Both groups received HPG and lacked additional carbon amendments.

## 3. Results and discussion

Sixty microbial communities were sequenced and analyzed through the experimental work described above. A full table of the most relatively abundant families in each of the 60 communities is provided in Supplementary Data Sheet 1. Sequences were analyzed to generate three types of data products: (1) alpha diversity values encompassing metrics of both richness and evenness (e.g., the Chao, Shannon, and Simpson statistics), which can be found in Supplementary Data Sheet 2; (2) beta diversity assessed through Aitchison distances (Supplementary Data Sheet 3) visualized in MDS ordination plots (described below); and (3) relative abundances of community members at the phylum (Supplementary Data Sheet 4) level. Below, we draw the reader’s attention to specific aspects of this substantial data set–while also conducting secondary analyses using primary data–in order to address the specific questions that motivated our study.

### 3.1. Method validation

#### 3.1.1. Constraining the effects of HPG addition

Correlation plots (Supplementary Figure 1) revealed a broadly consistent relationship between relative abundance data collected from cores incubated with and without HPG in both the 4- and 21-day experiments, suggesting that HPG does not substantially influence the distribution of microbial families. The “narrowing,” or heteroscedasticity, of the correlation plots at higher *x*- and *y*-axis values indicates that most of the variation potentially attributable to HPG presence or absence occurs with the low-abundance families. By focusing our analysis on high-abundance families, we minimize any confounding effects that may be associated with HPG addition.

We also sought to determine if incubation duration influenced the effects of HPG addition. After 4 days, the HPG-treated core (control treatment F) exhibited greater richness than the HPG-free control (control treatment E), but equivalent evenness (Supplementary Data Sheet 2). A similar trend was observed with the 21-day control study comparing samples B (with HPG) and A (HPG-free) (Supplementary Data Sheet 2). In an analysis of beta diversity, Aitchison distances were significantly different between the non-HPG control and the HPG control pooled communities within the 4-day incubated sediment cores (p_*EF*_ = 0.012), indicating that HPG presence had an effect on community structure. However, in a similar comparison of HPG addition, the 21-day incubations lacked significant difference (p_*AB*_ = 0.81), suggesting that the effect on community structure in the presence of HPG was transient. Together, these observations suggest that incubation time is a key determining factor in assessing the impact of experimental conditions. Aitchison distances between both controls that lack HPG (treatments A and E) are smaller than those between the HPG-incubated controls (treatments B and F), suggesting that HPG may contribute to differentiation in community composition across sediment horizons (Supplementary Data Sheet 3). When assessing relative proportions of lineages at the phylum level (Supplementary Data Sheet 4), we note that overall community structure is not substantially affected by HPG addition.

Prior studies have found negligible effects of HPG on the composition of sediment microbial communities (Hatzenpichler et al., 2014, 2016); however, a recent study reports that HPG triggered a stress response in *Synechococcus* (Michels et al., 2021). Our control experiments suggest that HPG addition could enhance community differentiation across sediment horizons, but that the effect on overall community structure is minimal in comparison to the effects of our experimental treatment, carbon amendment (discussed in further detail below). In this context, we focus on broad trends and abundant lineages rather than rare ASVs throughout the analyses that follow in order to attain meaningful results. In addition, by comparing carbon amendment incubations against the corresponding baseline of a parallel core treated with only HPG (e.g., C vs. B, D vs. B, and G vs. F), the contribution of exogenous carbon to microbial activity and community structure can be isolated.

#### 3.1.2. Incubation period influences microbial communities

**FIGURE 2:**
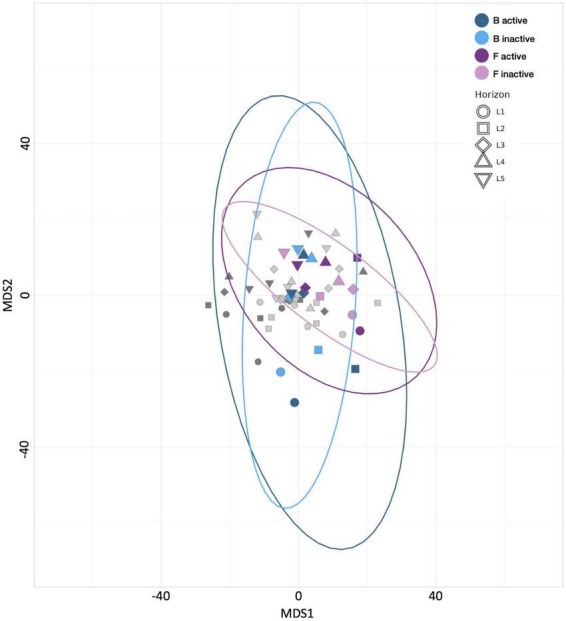
Multiple dimensional scaling (MDS) plot revealing the similarity between microbial communities according to Aitchison distance metrics. Blue points represent control treatment B (21 day incubated HPG controls) communities, purple points represent control treatment F (4 day incubated HPG controls) communities, and gray points represent communities from remaining treatments. Point shape represents horizon depth (*p* = 0.118; value determined for active communities).

No trends in community evenness were apparent, regardless of incubation time, depth, or activity. Among the anabolically active communities, the 4-day sample (control treatment F) exhibited greater richness across all depths than the 21-day sample (control treatment B; Supplementary Figure 2). This result could indicate that niche availability decreases with longer incubation periods. In the inactive communities, measures of richness revealed no clear correlation between diversity and either incubation time or depth. Beta diversity analyses and MDS plots indicated that control treatment F communities were less divergent than those from control treatment B (Figure 2); this trend suggests that the more extended incubation allowed communities to differentiate across sediment horizons. However, as noted with the assessments of HPG’s influence above, the distances between communities from these two control treatments are smaller than those observed between carbon-amended and control treatments (Data Sheet 2).

**FIGURE 3:**
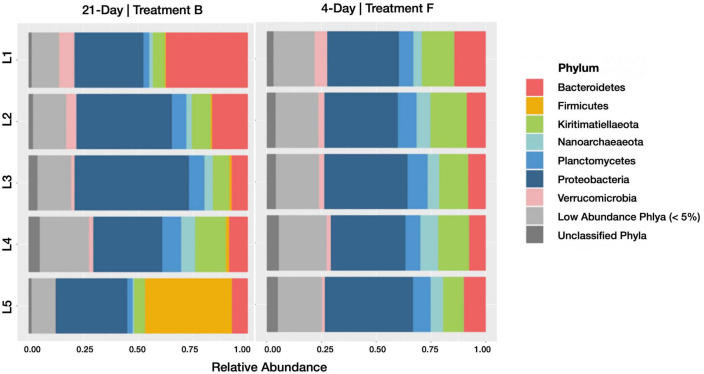
Barplots revealing the relative abundance of active phyla within each sediment horizon of the 21-day (control treatment B) and 4-day (control treatment F) cores. Communities with less than 5% relative abundance were pooled, as were ASVs of indeterminate phyla.

Relative abundances of taxonomic groups categorized at the phylum level showed more variation between horizons in the 21-day incubation than the 4-day incubation (Figure 3). In particular, we note a higher relative abundance of *Bacteroidetes* in the upper horizons of control treatment B (21-day incubation) active and inactive communities. The family *Cyclobacteriaceae* accounts for much of these differences, accounting for 8.3 and 9.4% of recovered sequences in control treatment B’s top horizon communities (active and inactive, respectively), and just 0.33 and 0.23% of control treatment F’s top horizon communities (active and inactive, respectively). Conversely, the high relative abundance of *Firmicutes* in the lowest horizon of control treatment B’s active community was not observed in the corresponding inactive community or either subset of control treatment F. It is difficult to separate the effects of duration-induced differentiation and natural spatial heterogeneity of salt marsh microbiomes, but because HPG incorporation is taxonomically agnostic (Hatzenpichler et al., 2020) any duration-induced increases in relative abundance should be captured only in the active community. Using this framework, the enrichment of *Bacteroidetes* in control treatment B (which was 1.6- and 2-fold more abundant than in control treatment F for the active and inactive communities, respectively) may be a result of spatial heterogeneity in the Berry Pool. Conversely, *Firmicutes* was enriched in the 4–5 cm horizon’s active fraction of control treatment B (18.9-fold more abundant than in control treatment F) but not in the inactive fraction (0.6-fold more/less abundant), meaning its higher relative abundance in control treatment B is more likely a result of incubation time. A different framework, in which differentiation is caused not by selective growth but by selective loss, would only be observed in the inactive community.

When assessing family-level relative abundances in the active communities of treatments B and F, only the *Cyclobacteriaceae* (*Bacteroidetes*) and *Streptococcaceae* (*Firmicutes*) results noted above passed our high-abundance (more than 5% relative abundance in any horizon), high-enrichment (at least 2x) threshold (Supplementary Figure 3). Two of the most prominent families in both treatments F and B were *Desulfobacteraceae* and *Desulfobulbaceae* (Supplementary Data Sheet 1), suggesting that a primary salt marsh ecosystem function of heterotrophic sulfate reduction was not dramatically affected by incubation duration. These results are consistent with a study that showed no statistically significant effects of HPG addition (5 or 50 μM) on sulfate reduction rates in cold seep sediments (Hatzenpichler et al., 2016).

The effects of both HPG addition and incubation time on community composition manifest as minor differences in the relative abundances of a few select lineages rather than as dramatic shifts in overall community structure, but the effects are difficult to fully disentangle. For this reason, we will restrict our analyses to conditions in which both incubation time and HPG addition are consistent between treatments being compared.

### 3.2. Effects of carbon amendments on microbial anabolic activity

The focus of our study was to evaluate the ways in which natural carbon (*Spartina* cordgrass) and anthropogenic carbon (diesel fuel) promotes or discourages anabolic activity among members of the salt marsh microbiome in distinct sediment horizons.

#### 3.2.1. Effects of natural carbon (*Spartina alterniflora*) amendment

In order to determine which organisms were anabolically active in the presence of naturally occurring organic carbon, sediment core samples treated with HPG and *Spartina* (treatments C and D) were compared to a control incubation that only received HPG (control treatment B). All three cores were incubated for 21 days, eliminating potential complications associated with the duration of the incubation experiments. The two *Spartina*-treated cores allowed us to track changes in active lineages during two distinct stages of lignocellulose breakdown: the core incubated with *Spartina* and HPG for 21 days (treatment C) provided insight into the immediate and cumulative response to added organic carbon, while the core that only received HPG for the final 4 days of the incubation (treatment D) revealed lineages responsive to later stages of decomposition.

Following 21 days of *in situ* incubation, measures of alpha diversity decreased in the two *Spartina*-exposed cores (treatments C and D) compared with the corresponding HPG control (control treatment B; Supplementary Figure 4). The distinction between treated and control communities diminished with horizon depth, indicating that the effects of the *Spartina* were observed most prominently in the top horizons over the course of the three-week experiment. This observation is consistent with the design of the experiment wherein the cordgrass was added on the top of the sediment and breakdown products were likely transported downward through bioturbation over time. Active communities were more diverse in later stages of *Spartina* degradation (treatment D) than earlier stages (treatment C). This result suggests that as the cordgrass breaks down, the number of available metabolic strategies increases as a greater range of taxa is able to utilize the lignocellulose constituents.

**FIGURE 4:**
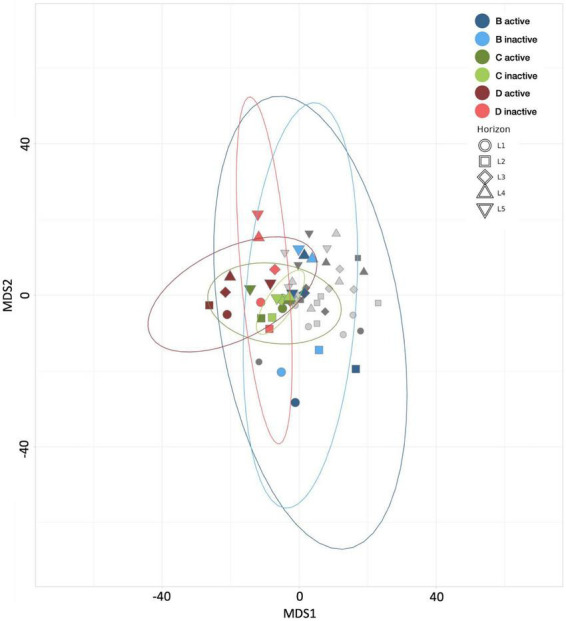
Multiple dimensional scaling (MDS) plot revealing the similarity between microbial communities according to Aitchison distance metrics. Blue points represent control treatment B (21 day incubated HPG controls) communities, green points represent treatment C (21 day *Spartina* and HPG incubations) communities, red points represent treatment D (21 day *Spartina* and 4 day HPG incubations) communities, and gray points represent communities from remaining treatments. Point shape represents horizon depth (pBD = 0.005, pBE = 0.008, pDE = 0.012; values determined for active communities).

The results of MDS analyses of the *Spartina* treatments and their respective controls (Figure 4) reflect the change in community composition in response to the breakdown of lignocellulose. While the control group communities from control treatment B are widely dispersed in MDS space, communities in each *Spartina* treatment are more tightly clustered, indicating that the addition of organic carbon drives shifts in beta diversity and greater homogeneity in both the active and inactive subsets. Moreover, communities from the *Spartina*-amended, 21-day HPG incubation (treatment C) exhibited the greatest similarities across horizons, while those that received HPG for only the final 4 days of incubation are less closely clustered. The relative positions of control treatment B, C, and D communities bolster the conceptualization of these three treatments as a time series, in which a *Spartina*-incubated core becomes increasingly distinct from its untreated counterpart over time.

Combined, our alpha and beta diversity results point to a two-phase community progression following *Spartina* introduction. Results of early-stage processes, as indicated by treatment C’s narrowed community richness and evenness, as well as a tighter clustering of its communities in MDS analyses, suggest that a restricted subset of taxa is able to perform the early breakdown of *Spartina*. Later stage results, as observed in treatment D (which received HPG in only the final 4 days of incubation) showed that alpha diversity values were greater, and MDS scores were more broadly distributed, than those of the early-stage analog (treatment C). These results indicate that a wider range of organisms is able to take advantage of subsequent stages of cordgrass decomposition. This pattern of decreasing diversity upon the addition of carbon, followed by increased diversity as degradation proceeds, has been observed previously in salt marsh sediments (Engel et al., 2017; Yu et al., 2022), as well as other systems such as grassland soil (Diamond et al., 2019) and kelp degradation (Feng et al., 2022).

In order to determine which organisms may be stimulated by the addition of *Spartina*, we investigated both consistently prevalent and differentially abundant anabolically active taxa in *Spartina*-treated cores. The former may include generalist lineages able to thrive with or without added cordgrass; the latter likely include specialist lineages that are most responsive to *Spartina* and/or its byproducts. We applied this framework to active communities at both the phylum level (in order to identify overarching trends at broader taxonomic resolution) and the family level (in order to pinpoint more specific metabolic strategies or lineages of particular ecological interest).

**FIGURE 5:**
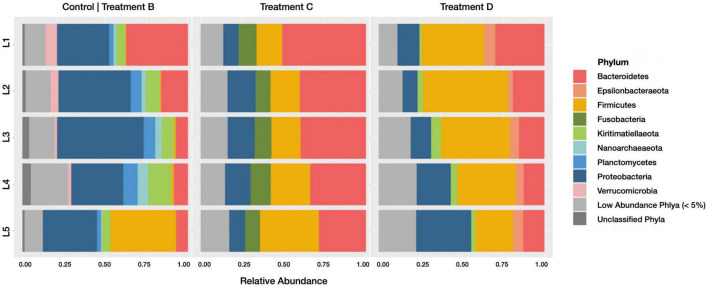
Barplots revealing the relative abundance of active phyla within each sediment horizon of the control core (group B), early stage *Spartina* treatment (treatment C), and late stage *Spartina* treatment (treatment D). Communities with less than 5% relative abundance were pooled, as were ASVs of indeterminate phyla.

When comparing phylum-level relative abundances within active communities (Figure 5), several lineages were classified as abundant (> 5% relative abundance in at least one horizon of one sample) upon the addition of *Spartina*. *Firmicutes* increased from 8.6% (±17.3% SD; across all horizons) in control group B to 22% (±8.2% SD) in treatment C and 37.6% (±10.4% SD) in the later stages of *Spartina* degradation captured by treatment D. This phylum includes many spore-forming organisms that may become active upon the addition of carbon (Galperin, 2013); its members are metabolically diverse and may play a role in sulfate consumption and organic carbon (e.g., acetate) formation in anoxic sediments (Roussel et al., 2015). *Fusobacteria* were anabolically active in all horizons (10.3 ± 1.37% SD) in treatment C but were present at low abundances in treatments B (1 ± 0.6% SD) and D (1.9 ± 0.6% SD). We interpret this pattern as an indication that this phylum, which is often implicated in fermentation processes (Ramezani et al., 1999; Kapatral et al., 2002), is a key player in the early stages (first 17 days) of *Spartina* breakdown. A study of tidal flat sediments reported an increase in *Fusobacteria* sequences and fermentation products after just 5 days of incubation with an organic carbon (cyanobacterial cells) amendment (Graue et al., 2012). *Epsilonbacteraeota* were most active in treatment D (representing 5.2 ± 1.4% SD of the active community across all horizons) compared with treatments B (0.08 ± 0.06% SD) and C (1.3 ± 0.5% SD), suggesting that this phylum grows well on secondary *Spartina* products. *Kiritimatiellaeota* are abundant in the active communities of the *Spartina*-free control treatment B (8.1 ± 3.6% SD), are sparsely represented in the integrated 21-day incubation (treatment C; 2.9 ± 0.7% SD) but return to a relative abundance of 9.1% (±4.1% SD) of the active community between days 17 and 21 of *Spartina* incubation (treatment D). This trajectory indicates that *Kiritimatiellaeota* may be outcompeted by early-stage lignocellulose degrading organisms but may rebound over the course of a few weeks. Other phyla, including *Verrucomicrobia*, *Planctomycetes*, and *Nanoarchaeota*, were only found in the *Spartina*-free incubation, while *Proteobacteria* decreased notably, from 38.3% (±9.3% SD) in control treatment B to 13.5% (±3.8% SD) in treatment C and 17.7% (±9.7% SD) in treatment D.

**FIGURE 6:**
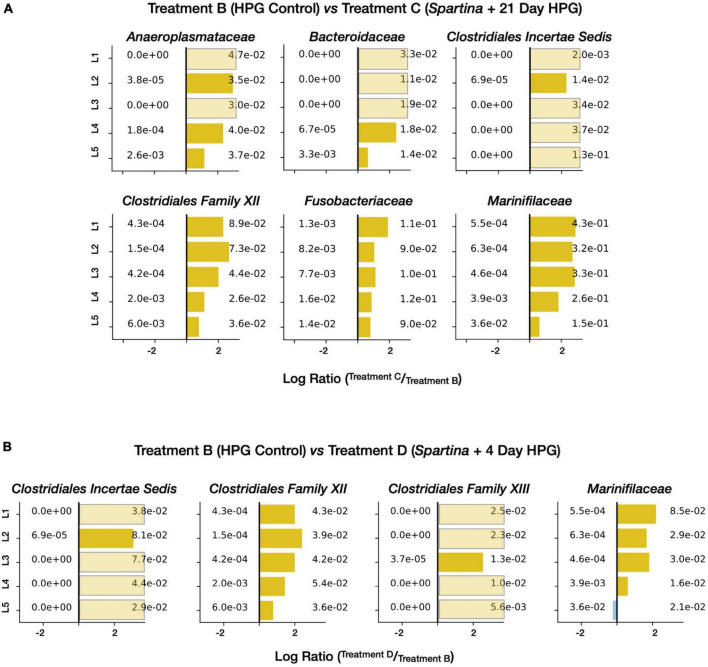
Enrichment figures depicting the relative abundance of select active families within each sediment horizon. Bars indicate the ratio between the control (control treatment B, blue) and treatment [treatment C **(A)** and treatment D **(B)**, yellow] communities. Faded bars represent ratios wherein the control group has a relative abundance of zero.

To attain a more taxonomically and metabolically specific sense of the organisms and processes stimulated by the addition of *Spartina*, we compiled a list of the most prominent families in the active subset of the 21-day HPG + *Spartina* incubation (treatment C; Supplementary Data Sheet 1). Six families were present at ± 1% relative abundance in all five horizons: *Marinifilaceae* (29.6 ± 10.3% SD), *Fusobacteriaceae* (10.2 ± 1.4% SD), the *Clostridia Family XII* (5.3 ± 2.6% SD), *Anaeroplasmataceae* (3.8 ± 0.6% SD), *Clostridiaceae 1* (3.4 ± 0.7% SD), and *Bacteroidaceae* (1.9 ± 0.8% SD). Remarkably, none of these families was consistently found above 1% relative abundance in the active communities of the HPG control (control treatment B), indicating that a substantial and specific shift in metabolic activity occurred following the addition of *Spartina*. In other words, specialist lineages appear to dominate during the early stages of cordgrass degradation while otherwise prominent taxa (such as the sulfate-reducing bacteria *Desulfobacteraceae* and *Desulfobulbaceae*) play smaller roles. “Enrichment” figures, which depict the ratio between the relative abundance of a given lineage in one community and its relative abundance in a reference community, are able to resolve horizon-specific activity patterns while controlling for pre-existing community structure. When applied to treatments B and C, this approach shows that five of the six families most responsive to initial *Spartina* exposure are most enriched in shallower sediment horizons (Figure 6A). This pattern suggests that the direct or indirect influence of 21 days of *Spartina* degradation is most felt in the top few centimeters of salt marsh sediments, while deeper horizons lacked equivalent exposure to carbon input.

A similar analysis of treatment D (later-stage *Spartina* degradation) revealed which families were most active 17–21 days after the addition of *Spartina*. Thirteen families exceed ± 1% of the community in all horizons (Supplementary Data Sheet 1); foremost among them were a clostridial “livecontrolB21” (6 ± 2.6% SD), *Clostridiales Incertae Sedis* (5.4 ± 2.4% SD), *Desulfovibrionaceae* (4.9 ± 2.4% SD), and the *Clostridia Family XII* (4.3 ± 0.7% SD). Of these 13 families, only one (*Spirochaetaceae*) met the same abundance threshold in control treatment B, and three (*Marinifilaceae*, *Fusobacteriaceae*, and *Family XII*) did so in treatment C. Enrichment plots of the most prevalent active families in treatment D (compared with control treatment B) show more consistent enrichment with sediment depth (Figure 6B), suggesting that *Spartina*-dependent activity between 17 and 21 days following the amendment is more distributed throughout the top five centimeters than activity integrated over the full 21-day period.

Taken together, our surveys of the most prevalent anabolically active families support the notion of compositionally and functionally distinct communities during both phases of *Spartina* breakdown that we tested. *Marinifilaceae* was prevalent in the active communities of both the integrated (treatment C) and later-stage (treatment D) *Spartina* incubations; this family includes aerobic, microaerophilic, and anaerobic members, many of which have been isolated from marine or salt marsh sediments (Wu et al., 2016; Vandieken et al., 2018; Park et al., 2019). One isolate from Antarctic marine sediment encodes xylanase, an enzyme capable of converting lignocellulose to simple sugar molecules (Han et al., 2019). In the sulfidic waters of the Black Sea, *Marinifilaceae* members accounted for ± 1% of the microbial community but were enriched to 78.7% (based on 16S rRNA gene reads) upon the addition of cellobiose (Yadav et al., 2020), the repeating molecular unit that comprises cellulose. Isolates from this enrichment were unable to reduce sulfate or nitrate, but rather fermented carbohydrate substrates to H_2_ and CO_2_ (Yadav et al., 2020). In a different study, metagenome-assembled genomes from the *Marinifilaceae* subgroup MF-2 enriched during incubations of deep-sea sediment with wood chips contained the genetic repertoire to oxidize lignin to methanol (Li et al., 2022). It thus seems possible that *Marinifilaceae* could be involved in multiple stages of lignocellulose breakdown, from the saccharification of the full polymer to lignin oxidation and cellulose fermentation. The representatives of this family detected in our samples may be most stimulated by the initial phases of lignocellulose breakdown (e.g., saccharification and lignin oxidation) given the substantial enrichment of active *Marinifilaceae* found in treatment C (Figure 6A).

Several other fermentative lineages were observed in the *Spartina*-amended incubations, and their preferential enrichment in distinct horizons of treatment C or D may provide a putative timeline of family-specific metabolic activity patterns. *Fusobacteriaceae* were most prevalent in treatment C, accounting for 10.2% (±1.4% SD) of the active population across all depths (Supplementary Data Sheet 1) and were particularly enriched in the top horizon (Figure 6A). *Fusobacteriaceae* are microaerophilic or obligate anaerobes that ferment carbohydrates [including cellulose (Van Gylswyk, 1980)] or amino acids into a range of organic acids (Olsen, 2014); one lineage exclusively ferments quinic and shikimic acids (Brune et al., 2002), which are abundant in vacuoles of vascular plants such as cordgrass (Yoshida et al., 1975). These patterns suggest that electron acceptor limitation occurred even in some areas of the uppermost horizon within a few weeks of *Spartina* introduction, and that *Fusobacteriaceae* may be among the first fermenters to take advantage. *Anaeroplasmataceae* were both abundant (Supplementary Data Sheet 1) and enriched (Figure 6A) in all horizons of treatment C. This family is a member of the *Mollicutes*, a class of microorganisms distinguished by their small size, lack of cell wall (Razin, 2006), and range of parasitic, commensal, and free-living lifestyles (Razin et al., 1998; Skennerton et al., 2016). *Anaeroplasmataceae* require sterols for growth (Brown et al., 2015), which may be derived in large part from *Spartina* (Lee et al., 1980). Their fermentative metabolism can generate a range of products including organic acids, ethanol, CO_2_, and H_2_ (Robinson and Freundt, 1987).

Two families of the class *Clostridia* were also present at high abundance (and enriched relative to the HPG-incubated control; Figure 6A) in treatment C: *Clostridia Family XII* and *Clostridiaceae_1*. While the *Clostridia* class as a whole is typically associated with fermentative metabolisms (Mead, 1971; O’Brien and Ljungdahl, 1972; Winter et al., 1987), and *Clostridia Family XII* may include fermenters of complex organics, some members of the *Clostridiaceae_1* family have been found to oxidize hydrogen with molecular oxygen (Stadtman and Barker, 1949; Santana, 2008); the prevalence of this family in our active subset of treatment C (3.43 ± 0.67% SD) is consistent with initial incubation conditions when oxic niches were most likely to be present [The H_2_ may be sourced from fermenters such as *Marinifilaceae* and *Anaeroplasmataceae*, and could be oxidized not only by *Clostridia* representatives, but also several other taxa including members of the *Bacteroidota*, *Chloroflexota*, *Planctomycetota*, and *Verrucomicrobiota* phyla, as shown by Bay et al. (2021) in a range of soil environments]. Another *Clostridia* family, *Lachnospiraceae*, was found in both *Spartina* incubations at lower relative abundances (0.75 ± 0.42% SD for treatment C; 1.20 ± 2.15% SD for treatment D); this family can ferment a narrow range of substrates including pectin, a component of plant biomass (Doran-Peterson et al., 2008; Sorokin et al., 2012).

**FIGURE 7:**
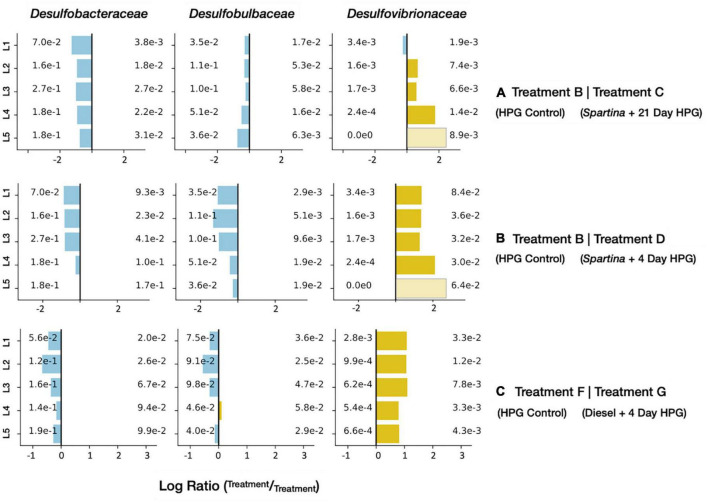
Enrichment figures depicting the relative abundance of active SRB families within each sediment horizon. Bars indicate the ratio between the control groups (blue) and their respective treatment group (yellow) communities. Faded bars represent ratios wherein the control group has a relative abundance of zero.

The 17–21 day period of *Spartina* breakdown captured by the treatment D incubation revealed a distinct shift in anabolically active microorganisms measured both at the level of the full community (see above) and specific lineages. In particular, select sulfur-cycling lineages were detected at high relative abundances in comparison with the HPG control communities recovered from control treatment B. Sulfate-reducing bacteria (SRB) can use a wide range of electron donors, including many products of fermentative metabolism such as organic acids and H_2_ (Hansen, 1993) that may have been mobilized during the initial phase of the *Spartina* incubation. While three families of SRB–*Desulfobacteraceae*, *Desulfobulbaceae*, and *Desulfovibrionaceae*–were common in all control and amended treatments, only the latter was enriched upon the addition of *Spartina* (Figure 7). In a study that added ^13^C-labeled cyanobacterial biomass to tidal flat sediments in Germany, *Desulfovibrio* was the most ^13^C-enriched SRB lineage as sulfate concentrations decreased and primary fermentation products became available (Graue et al., 2012). *Desulfovibrionaceae* oxidize organic substrates incompletely to acetate and use H_2_ as an electron source (Voordouw, 1995); they have been shown to exhibit higher growth rates than other sulfate reducers (Barnes and Mead, 1986; Widdel, 1986), particularly under sulfate-limiting conditions (Laanbroek et al., 1984). A different sulfur-reducing family, *Sulfurospirillaceae*, was present at 3.2% (±0.8% SD) relative abundance in treatment D active communities across all horizons; this lineage similarly oxidizes H_2_ or organic acids (e.g., formate) but reduces O_2_ or elemental sulfur instead of sulfate (Finster et al., 1997). Their prevalence throughout all five horizons in treatment D (Supplementary Data Sheet 1) suggests that elemental sulfur, perhaps generated by incomplete oxidation of sulfide, is the more likely electron acceptor.

Some *Clostridia* families were also enriched in treatment D communities. *Family XII* remained prevalent, though less so than in treatment C. *Syntrophomonadaceae* accounted for 3.7% (±1.2% SD) of the active communities across all horizons and were most abundant between 1 and 4 cm deep. Members of this family have been shown to oxidize carboxylic acids (a likely product of earlier fermentation) to H_2_ or formate and are often found in close association with hydrogenotrophic organisms that keep product concentrations low to enhance the reaction’s energetics (Sobieraj and Boone, 2006). The ASVs categorized at the family level as *Clostridiales Incertae Sedis* were also more prevalent in treatment D and had closest genus-level matches to *Dethiosulfatibacter*. This genus is able to couple the reduction of intermediate sulfur compounds (thiosulfate and elemental sulfur, but not sulfate) with the partial oxidation of amino acids and subsequent fermentation to acetate and H_2_ (Takii et al., 2007). *Dethiosulfatibacter* was initially isolated from a coastal sediment co-culture with a *Desulfovibrio* strain, whose consumption of the fermentation product H_2_ enhanced both organisms’ growth rates (Takii et al., 2007).

Striking microbial aggregates known as “pink berries” were observed along the sediment surface of Sippewissett’s tide pools. The syntrophic berries are made up of a sulfate-reducing phylotype most closely related to *Desulfobulbaceae* and purple sulfur bacteria of the family *Chromatiaceae* (Wilbanks et al., 2014). Both of the pink berries’ dominant constituents were more prevalent in the active subset of the HPG control (control treatment B) than the *Spartina*-treated incubations (treatments C and D). Neither family was stimulated by the addition of *Spartina*; this finding is consistent with their phototrophy-powered metabolism and suggests that the pink berries experience minimal metabolic impact from the introduction of cordgrass carbon.

Some analogous studies of lignocellulose breakdown identified different active taxa making similar putative metabolic contributions as those described above. For example, a study of California salt marsh mesocosms attributed lignocellulose breakdown to *Spirochaeta* and consumption of degradation products to *Kaniella* and *Desulfosarcina* (Darjany et al., 2014). An investigation of UK salt marsh sediments found abundant lignocellulose-degrading enzymes produced by families such as *Prolixibacteraceae*, *Flavobacteriaceae*, *Vibrionaceae*, *Cellvibrionaceae*, *Saccharospirillaceae*, *Alteromonadaceae*, and *Cytophagaceae* (Leadbeater et al., 2021). Esterase enzymes were also prevalent, revealing a metabolic decoupling of lignin and cellulose breakdown pathways (Leadbeater et al., 2021). These results suggest that salt marsh sediments from geographically distinct areas possess a wide range of species exhibiting substantial functional redundancy. The specific taxa that predominate are likely dependent upon localized geochemistry or physicochemical parameters.

**FIGURE 8:**
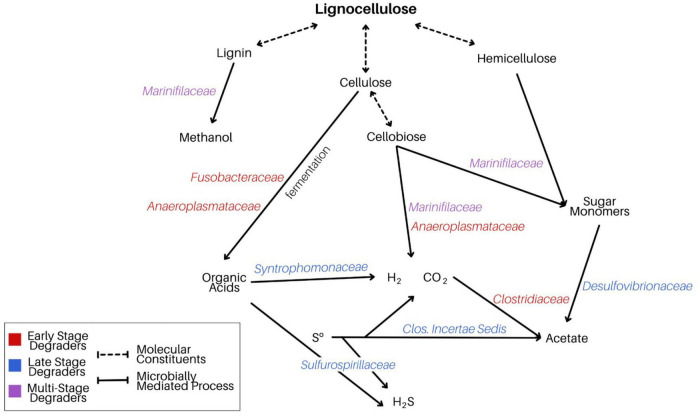
Schematic diagram demonstrating our proposed model of microbially mediated *Spartina* breakdown in LSSM. We attribute the degradation of the cordgrass’s three primary components to a multi-stage process driven by a range of taxa poised to respond to a variety of downstream *Spartina* metabolites.

The data presented here offer a two-stage perspective of the microorganisms engaged in *Spartina* breakdown, revealing an initial narrowing in diversity associated with lignocellulose metabolism, followed by a broadening range of active taxa as primary fermentation products bolster sulfur-cycling heterotrophs. By identifying specific lineages that were enriched in the active communities during these distinct phases, a perspective of *Spartina*-stimulated microbial activity emerges (Figure 8). Lignocellulose consists of three primary constituents (lignin, cellulose, and hemicellulose), each of which can be oxidized to a range of products by versatile lineages such as *Marinifilaceae* and more specialized fermenters like *Fusobacteriaceae* or *Anaeroplasmataceae*. After two and a half weeks, these initial products are further oxidized through metabolisms that interface with the sulfur cycle, performed by lineages such as *Desulfovibrionaceae* and *Sulfurospirillaceae*. The dynamics illuminated by our BONCAT experiments reveal complex, multi-stage links between *Spartina* metabolism, a range of fermentation approaches, and sulfur metabolism, presenting a perspective of successional dynamics following the introduction of *Spartina*.

#### 3.2.2. Effects of anthropogenic carbon (diesel) amendment

In order to evaluate the impact of anthropogenic organic carbon on microbial community activity, sequence data from diesel treated sediment (treatment G) and the corresponding HPG control (control treatment F) were compared. Both the control and treatment cores were incubated *in situ* in the LSSM Berry Pool for 4 days to eliminate any complications due to incubation duration (see section “3.1.2. Incubation period influences microbial communities”).

Alpha diversity analyses of the anabolically active communities reveal that both richness and evenness decrease with the addition of diesel (Supplementary Figure 5); this effect is most pronounced within the top three horizons (0–3 cm sediment depth). It is challenging to separate the availability of diesel (which was added at the sediment-water interface) from the presence of diesel-utilizing microbes (which may be preferentially present in the top horizons). However, given the visual observation of diesel remaining in the overlying water phase at the end of the four-day incubation (data not shown), it is very likely that the upper sediment horizons experienced a higher diesel exposure than deeper horizons. Nonetheless, it is possible that the deeper horizons we analyzed still experienced some alteration in the form of low diesel concentrations or more diffusible metabolic byproducts from upper-horizon activity. It is also possible that overlying diesel affected gas exchange dynamics, potentially causing an upward migration of the oxic-anoxic interface [which is already captured in our top horizon (0–1 cm) samples; Larsen et al., 2015; Salman et al., 2015].

**FIGURE 9:**
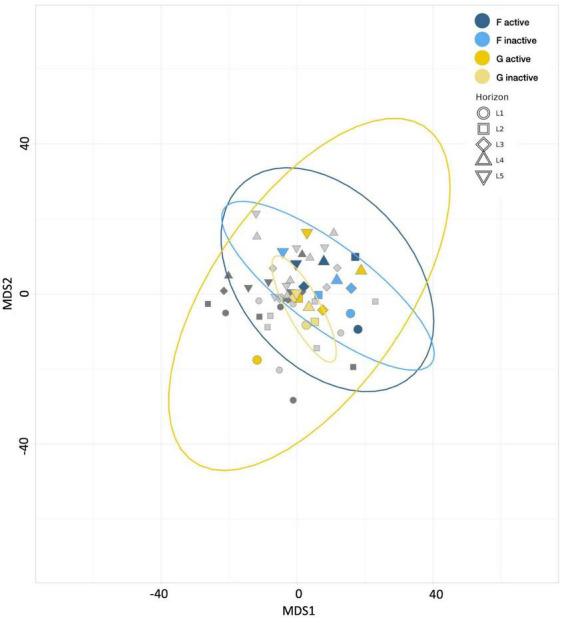
Multiple dimensional scaling (MDS) plot revealing the similarity between microbial communities according to Aitchison distance metrics. Blue points represent control treatment F (4 day incubated HPG controls) communities, yellow points represent treatment G (4 day diesel HPG incubations), and gray points represent communities from remaining treatments. Point shape represents horizon depth (*p* = 0.028; value determined for active communities).

Aitchison distances were used to generate an MDS plot (Figure 9), which revealed that the communities from active and inactive subsets of the diesel treatment were significantly different (*p*-value: 0.001) from those in the HPG-only control group. The active communities exhibited a wider spread in inter-horizon community composition than their inactive counterparts, with notable separation between the top horizons and lower two horizons. These results are consistent with the alpha diversity metrics, which depict greater variation in the uppermost horizons of the treatment cores.

**FIGURE 10:**
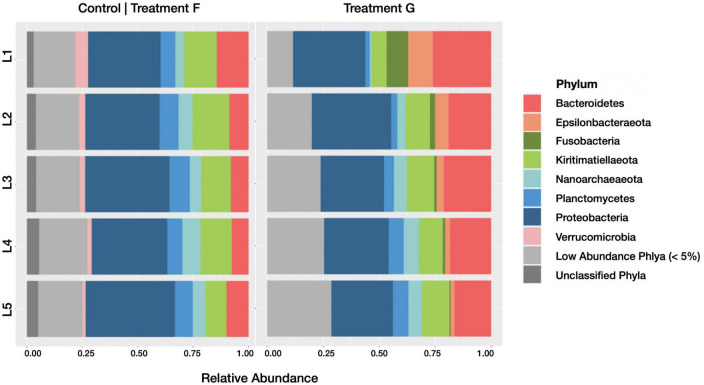
Barplots revealing the relative abundance of active phyla within each sediment horizon of the control core (control treatment F) and diesel treatment (treatment G). Communities with less than 5% relative abundance were pooled, as were ASVs of indeterminate phyla.

The relative abundance of taxa present in the active communities of the treatments G and F were compared to assess changes associated with the presence of diesel. We found that, at the phylum level (Figure 10), *Planctomycetes* decreased (from a relative abundance of 8.12 ± 1.09% SD across all horizons in control treatment F to 4.55 ± 2.24% SD in treatment G), while *Fusobacteria* (0.06 ± 0.10% SD to 2.94 ± 3.80% SD), *Epsilonbacteraeota* (1.38 ± 0.97% SD to 4.80 ± 3.80% SD), and *Bacteroidetes* (9.75 ± 2.76% SD to 20.22 ± 3.67% SD) all increased with the diesel amendment. As observed with the broader measures of active community diversity, these changes were most evident in the top sediment horizon.

Diesel consists of hydrocarbons of variable size and structure that can have toxic effects on multiple members of a microbial ecosystem (van Dorst et al., 2014). The mixture consists of both straight run hydrocarbons (unprocessed) and catalytically cracked hydrocarbons (smaller molecules derived from longer molecules in the interest of refining the fuel for industrial use). Diesel is primarily composed of paraffins (long chain alkanes), polycyclic aromatic hydrocarbons (PAHs), and hydrodesulfurized middle distillate (Gad, 2005). PAHs include large, insoluble, nonpolar molecules that accumulate in fine-grained sediments, as well as smaller molecules that are more metabolically accessible. Hydrodesulfurized middle distillate is processed such that sulfur is removed from the straight run stream of hydrocarbons. Overall, diesel’s elemental composition is ±87% carbon, ±13% hydrogen, and less than 1% sulfur and nitrogen (Ahmed et al., 1999); its presence in our incubations may provide a range of oxidizable carbon molecules but a relative lack of key nutrients (N and P species) and exogenous electron acceptors.

**FIGURE 11:**
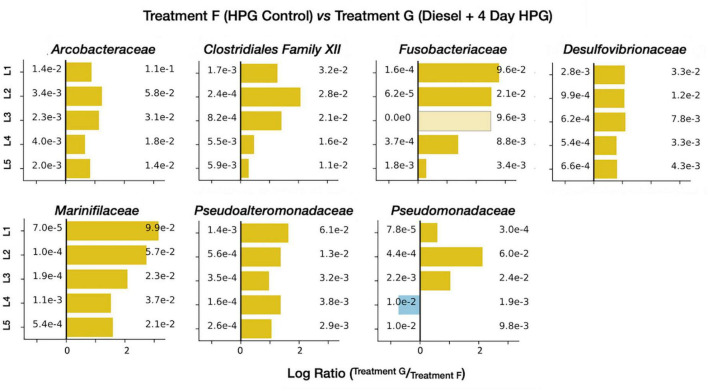
Enrichment figures depicting the relative abundance of select, active families within each sediment horizon. Bars indicate the ratio between the control (group F, blue) and treatment (group G, yellow) communities. Faded bars represent ratios wherein the control group has a relative abundance of zero.

The anabolically active diesel-treated community (treatment G) had nine families with an average relative abundance of ± 1% across all five horizons (Supplementary Data Sheet 1). Of these families, *Arcobacteraceae*, *Marinifilaceae*, and *Clostridia Family XII* were not found above this 1% threshold in the diesel-free HPG control (control treatment F). Enrichment figures (Figure 11) directly comparing relative abundances between the treatments at each horizon depth indicated that four additional families were preferentially identified in the diesel-treated sample: *Fusobacteriaceae*, *Desulfovibrionaceae*, *Pseudoalteromonadaceae*, and *Pseudomonadota*.

The long-chain alkanes and PAHs found in diesel are molecular classes composed of many (often poorly constrained) compounds; this range of organic carbon reactants may be favored by specific community members involved in distinct stages of degradation. Many of the abundant and enriched families in treatment G are consistent with a microbial community, derived from a contaminated industrial site in Germany, that degraded benzene (an aromatic hydrocarbon). Through stable isotope probing, Starke et al. (2016) proposed an initial benzene fermentation step (mediated by *Clostridiales*) generating hydrogen and acetate, products that were then oxidized by *Desulfobacterales* and *Campylobacterales*, respectively. In diesel-impacted LSSM sediment, our data, in combination with previous studies (Mead, 1971; O’Brien and Ljungdahl, 1972; Van Gylswyk, 1980; Winter et al., 1987; Brune et al., 2002; Olsen, 2014; Yadav et al., 2020), suggest that *Marinifilaceae*, the clostridial *Family XII*, and *Fusobacteriaceae* are the most abundant lineages conducting the initial fermentation; the resulting organic acids may be consumed by *Arcobacteraceae* (a family within the *Campylobacterales*), and hydrogen may be used to power sulfate reduction among the *Desulfobulbaceae*, *Desulfobacteraceae*, and *Desulfovibrionaceae*. Of these three SRB taxa, all were abundant, but only *Desulfovibrionaceae* was enriched in the active subset of treatment G compared with the HPG control (Figure 7).

Other bacterial families were stimulated by diesel addition as well. The gammaproteobacterial families *Pseudoalteromonadaceae* and *Pseudomonadaceae* were enriched in the diesel-treated core (Figure 11); both taxa have been implicated in PAH degradation. *Pseudoalteromonadaceae* was abundant in Arctic seawater mesocosms that degraded naphthalene (Bagi et al., 2014), and isolates from the North Sea grew on PAHs including fluorene, phenanthrene, and anthracene, as well as C_10_–C_20_ alkanes (Chronopoulou et al., 2015). The *Pseudoalteromonas* genus was abundant in oil-impacted surface water (Redmond and Valentine, 2012) and water column (Gutierrez et al., 2013) samples from the Gulf of Mexico, particularly after alkanes had been consumed and aromatic molecules remained (Dubinsky et al., 2013). Species of this lineage have also been isolated from phenanthrene, chrysene and naphthalene-enrichment cultures of marine sediments (Melcher et al., 2002; Hedlund and Staley, 2006; Cui et al., 2008). *Pseudomonadaceae* have been detected at hydrocarbon contaminated sites (Aitken et al., 1998; Romero et al., 1998) and cultured representatives can degrade a range of PAHs (Mueller et al., 1990; Rockne et al., 2000). In a study of Baltic Sea sediment, an elevated expression of PAH-ring hydroxylating dioxygenase genes was attributed to *Pseudomonas* members, which were particularly responsive to added pyrene and phenanthrene (Iburg et al., 2020).

Many of the lineage-specific effects described above were more prevalent in the top sediment horizons than in the lower core sections (Figure 11). This was particularly true for the putative fermentative families (e.g., *Marinifilaceae*, *Fusobacteriaceae*, and *Pseudoalteromonadaceae*), which may indicate that immiscible, high-molecular weight diesel components remain at the sediment-water interface while breakdown products like acetate and hydrogen diffuse more easily through the sediment.

Intriguingly, obligate hydrocarbonoclastic bacteria (OHCB) that are commonly associated with oil degradation–such as the genera *Alcanivorax*, *Cycloclasticus*, and *Thalassolituus*–were only found in very low abundances in our diesel-incubated samples. This observation could be due to their low relative abundance in the sediment inoculum, their more specialized niches relative to the PAH- and hydrocarbon-degrading generalists we did detect (Chronopoulou et al., 2015), the comparatively higher concentration of PAHs present in crude oil, and/or the anti-biofilm activity of lineages such as *Pseudoalteromonas* (Dheilly et al., 2010; Klein et al., 2011), which may inhibit OHCB attachment to diesel-impacted sediment grains.

**FIGURE 12:**
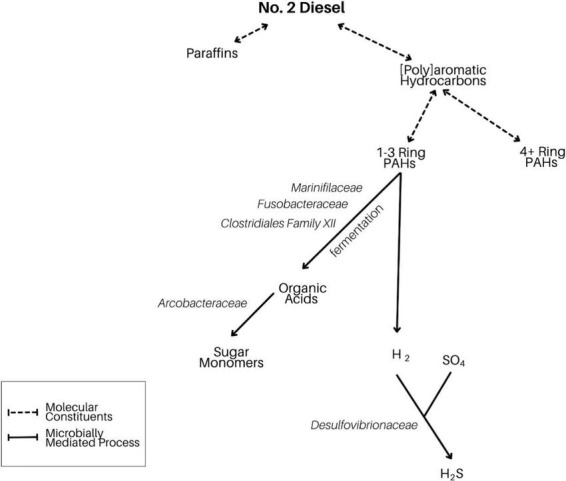
Flowchart demonstrating our proposed model of microbially mediated diesel breakdown in LSSM. Here, we focus on the degradation of small aromatic hydrocarbons, which is likely driving the community shift in our anthropogenic experiment.

Based on the taxa enriched in the different fractions of our incubations, we report evidence for select diesel-degrading metabolisms (Figure 12). In particular, fermentation of small PAHs by lineages such as *Marinifiliaceae*, *Fusobacteriaceae*, and the clostridial *Family XII* generates electron donors that may fuel heterotrophic (*Arcobacteraceae*) and sulfate-reducing (*Desulfovibrionaceae*) organisms. The less pronounced appearance of organisms involved in the metabolism of larger diesel-derived molecules including paraffin and large PAHs could be due to both their metabolic intractability and the brief duration of our experimental incubations.

### 3.3. Role of carbon source on community structure and activity

As demonstrated in our method validation analyses, incubation time can influence microbial community structure. While we can thus not directly compare the results from the *Spartina*-treated cores (C, D) with the diesel-treated core (G), by focusing on the trends illuminated through comparison of treated cores with their appropriate HPG controls, some compelling trends have emerged.

One commonality between the *Spartina*- and diesel-treated groups is reflected in the community-wide diversity trends. Regardless of its source, added carbon narrowed the alpha and beta diversity according to all metrics (Supplementary Data Sheets 2, 3). The latter stage of the *Spartina* experiment indicated a rebound in measures of diversity, and while the diesel experiment only had a 4-day timeframe, the metabolites that resulted from this carbon amendment likely stimulated a wider range of organisms capable of consuming fermentation products. Taken together, these observations suggest that abundant organic carbon enriches select lineages within a community that are poised to respond to the specific additive, potentially at the expense of the short-term biodiversity. As degradation proceeds and later-stage metabolites are produced, cross-feeding results, in which metabolites resulting from one nutrient source support downstream metabolic activity from additional lineages.

Models of cross-feeding demonstrate the ability of a single nutrient to sustain diverse communities. Dal Bello et al. (2021) showed that complex, high molecular weight molecules, when added on their own, support a more diverse range of microbial constituents than simple organics. Furthermore, each additional amendment resulted in a disproportionately smaller increase to the microbial diversity of the community. These results are consistent with our observation that *Spartina*-derived lignocellulose supported a more diverse community as it was broken down into smaller molecules. In fact, Dal Bello et al. (2021) found that cellulose and its downstream constituents supported the largest and most varied community of microbes when compared with 15 other sources of organic carbon.

We also noticed a prevalence of anabolically active fermentative lineages in both carbon amendment experiments. *Marinifilaceae*, *Fusobacteriaceae*, and members of the *Clostridia* class were enriched in the presence of both *Spartina* and diesel. While the specific lineages that respond to organic carbon are modulated by the pre-existing community at LSSM, this functional niche is common in estuarine ecosystems where anoxic conditions are found within a few millimeters of sediment depth and exogenous electron acceptors may be limited due to the activity of sulfate-reducing bacteria (SRB).

Sulfate-reducing bacteria (SRB) are ubiquitous in LSSM and were found at high relative abundances across all of our incubation treatments. The three families *Desulfobacteraceae*, *Desulfobulbaceae*, and *Desulfovibrionaceae* accounted for a substantial proportion of reads in nearly all samples, regardless of carbon amendment, sediment depth, or degree of anabolic activity. However, only the *Desulfovibrionaceae* family was enriched in the presence of amended carbon when compared with the corresponding HPG control condition. Differential responses among these three families have been described previously. Laanbroek and Pfennig (1981) and Laanbroek et al. (1984) have conducted several studies investigating the underlying drivers of competition between *Desulfobacter* spp., *Desulfobulbus* spp., and *Desulfovibrio* spp. This work highlighted the ability of each SRB lineage to preferentially utilize specific energy sources: *Desulfobacter* spp. metabolizing acetate, *Desulfobulbus* spp. metabolizing propionate, and *Desulfovibrio* spp. metabolizing hydrogen. By testing a range of conditions, *Desulfovibrio* spp. were found to outcompete *Desulfobacter* spp. and *Desulfobulbus* spp. in environments limited by sulfate (Laanbroek et al., 1984). Although our work did not alter sulfate concentrations, the addition of either excess natural or anthropogenic carbon likely lowered sulfate availability on localized scales, potentially establishing it as a limiting reactant. As a result, *Desulfovibrionales* would have been well positioned to outcompete other SRB lineages. By adding an excess of carbon to largely anoxic sediments, we believe that all sulfate reduction activity increased, but that sulfate was reduced first by *Desulfovibrionaceae*, leading to the enrichment of this family in both of our amendment experiments.

## 4. Conclusion

Using substrate analog probing during the *in situ* incubation of salt marsh sediment with two types of organic carbon, we traced the substantial effects of natural and anthropogenic carbon on the functional diversity of the resident microbial communities. Alpha and beta diversity metrics revealed a decrease in community diversity in response to both natural and anthropogenic organic carbon amendments. A window into secondary-stage breakdown of lignocellulose suggested that the metabolites from earlier stages of degradation supported a broader, more diverse microbial community. In both amendment experiments, microbial communities were most impacted in upper sediment horizons (0–2 cm), suggesting that microbial communities in deeper horizons (2–5 cm) are insulated from chemical inputs at the water column-sediment interface.

*Marinifilaceae* was particularly prevalent in the active communities of both carbon-amendment experiments, suggesting this family is metabolically flexible and can thus respond quickly to the availability of complex organic molecules. Fermentation was also implied to be a prominent metabolic function in the presence of both *Spartina* and diesel, likely attributed to *Marinifilaceae*, *Fusobacteriaceae*, and families within the order *Clostridiales*. SRB were present at high abundance in all samples, but *Desulfovibrionaceae* were most enriched in the active communities of all carbon treatments; this result could be due to this family’s ability to out-compete other SRB in sulfur limited conditions generated by organic carbon addition.

Future experiments will build upon our experimental foundation and further clarify the temporal and spatial details of carbon degradation in salt marsh-hosted microbial communities. Longer term experiments would reveal community and activity-associated changes as degradation processes continued beyond a few weeks. To this end, the use of BONCAT could be expanded to include a dual-tagging approach wherein multiple non-canonical amino acids are used to monitor cellular activity at distinct time points throughout the course of the experiment. Alternate carbon sources, or dual carbon source experiments, would reveal the microbial response to other carbon inputs, as well as phylogenetically-resolved preferences for certain carbon sources. Geochemical measurements would provide further insight regarding the overall nutrient flux in response to carbon amendments and would clarify the conditions most amenable to distinct SRB families.

Salt marshes are highly productive habitats with rapid carbon cycling dynamics. Microbial communities play a vital role in these processes, and the work presented here offers a temporally- and spatially-resolved understanding of lineage-specific patterns in metabolic activity under distinct carbon-loading regimes. By linking metabolic activity with taxonomic identity under distinct environmental conditions, we have pinpointed lineages that may be responsible for carbon cycling and bioremediation capabilities. This research thereby offers a compelling opportunity to ultimately protect and harness the regenerative metabolic processes of salt marsh sediments.

## Data availability statement

The datasets presented in this study can be found in online repositories. The names of the repository/repositories and accession number(s) can be found below: https://www.ncbi.nlm.nih.gov/, PRJNA973662.

## Author contributions

RS, RH, PG, and JM designed this study. RS, RH, and JM conducted field work. RS performed FACS and gene amplicon sequencing experiments. EF, RS, MS, and JM analyzed the data. RH was responsible for funding. PG, RH, and JM supervised the project. EF and JM wrote the manuscript. All authors contributed to the article, edited the manuscript, and approved the submitted version.
