# Supplementary figures and images for: Natural and anthropogenic carbon input affect microbial activity in salt marsh sediment
Source: Front Microbiol. 2023 Sep 7;14:1235906. doi: 10.3389/fmicb.2023.1235906 (PMC10512730; doi:10.3389/fmicb.2023.1235906)

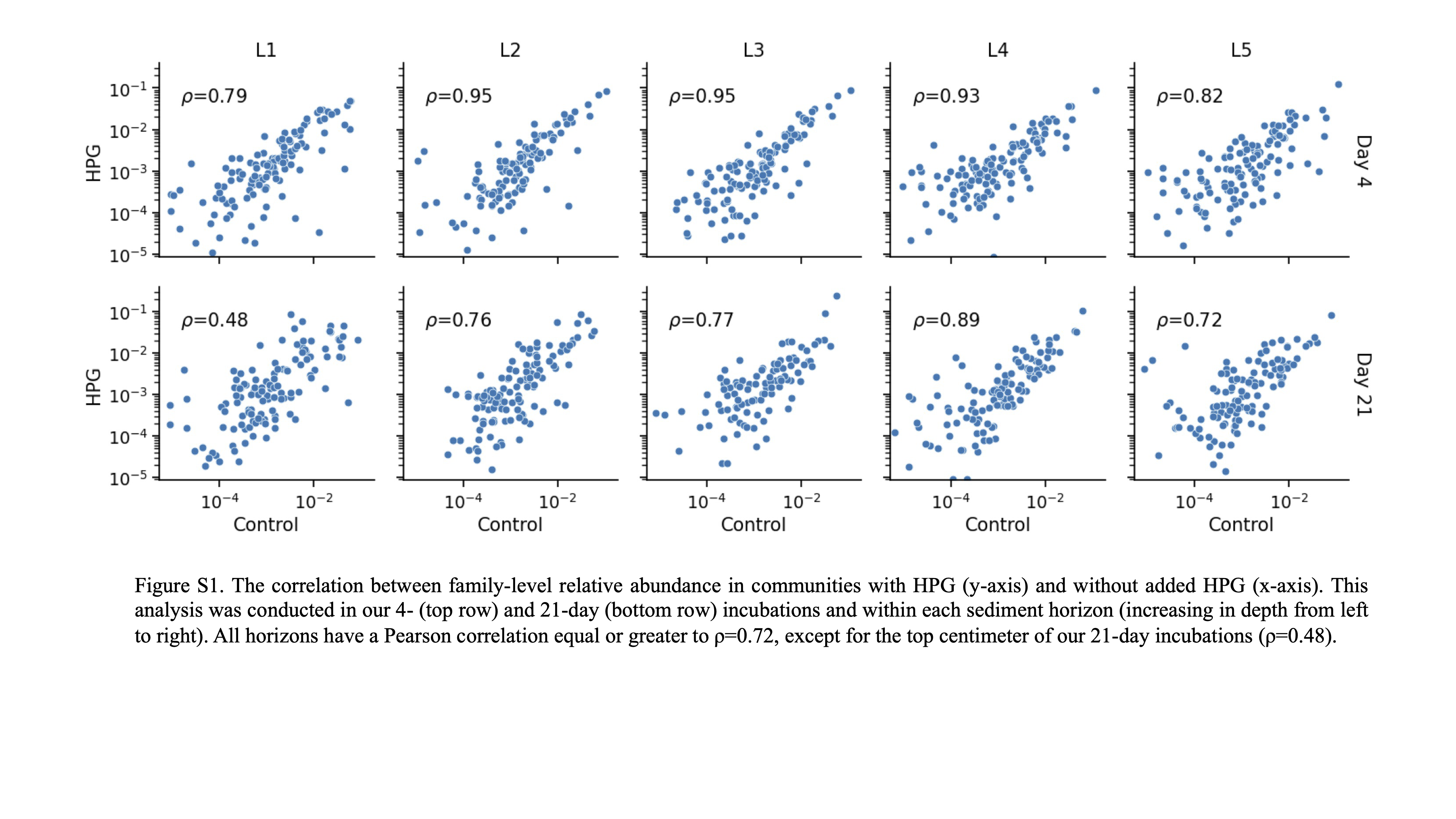

Supplement: Supplementary file 5 [file Image_1.TIFF]

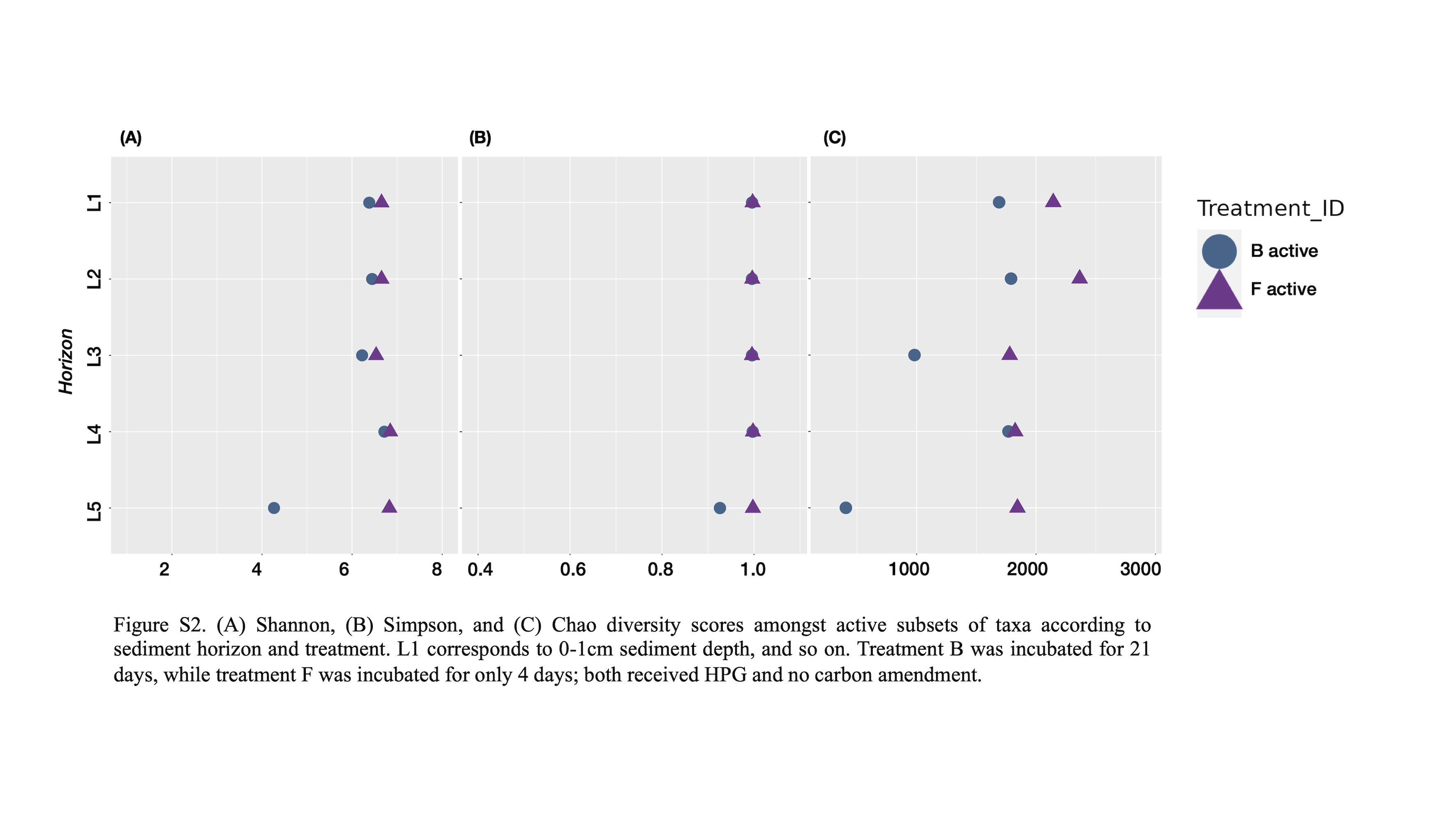

Supplement: Supplementary file 6 [file Image_2.TIFF]

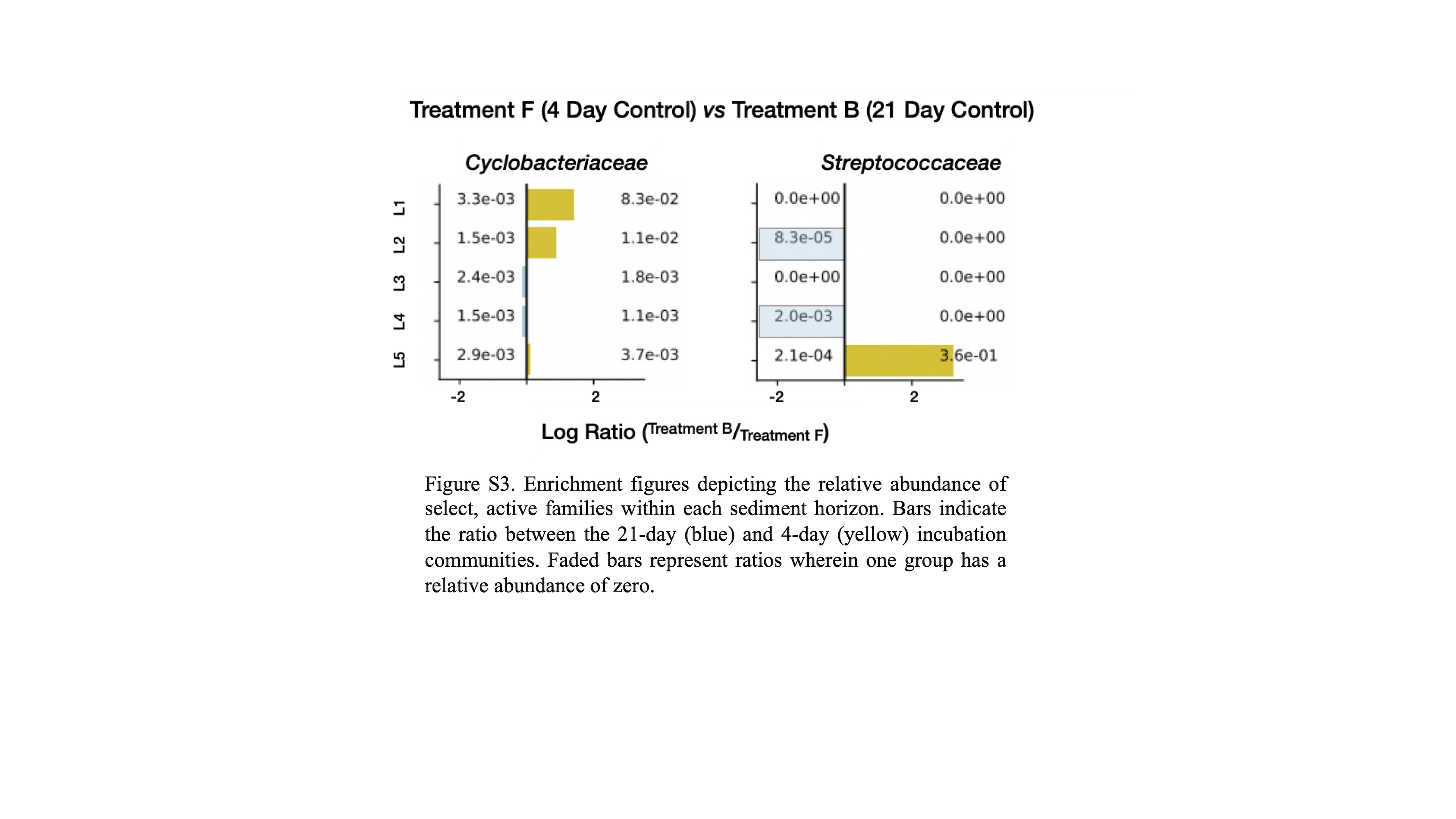

Supplement: Supplementary file 7 [file Image_3.TIFF]

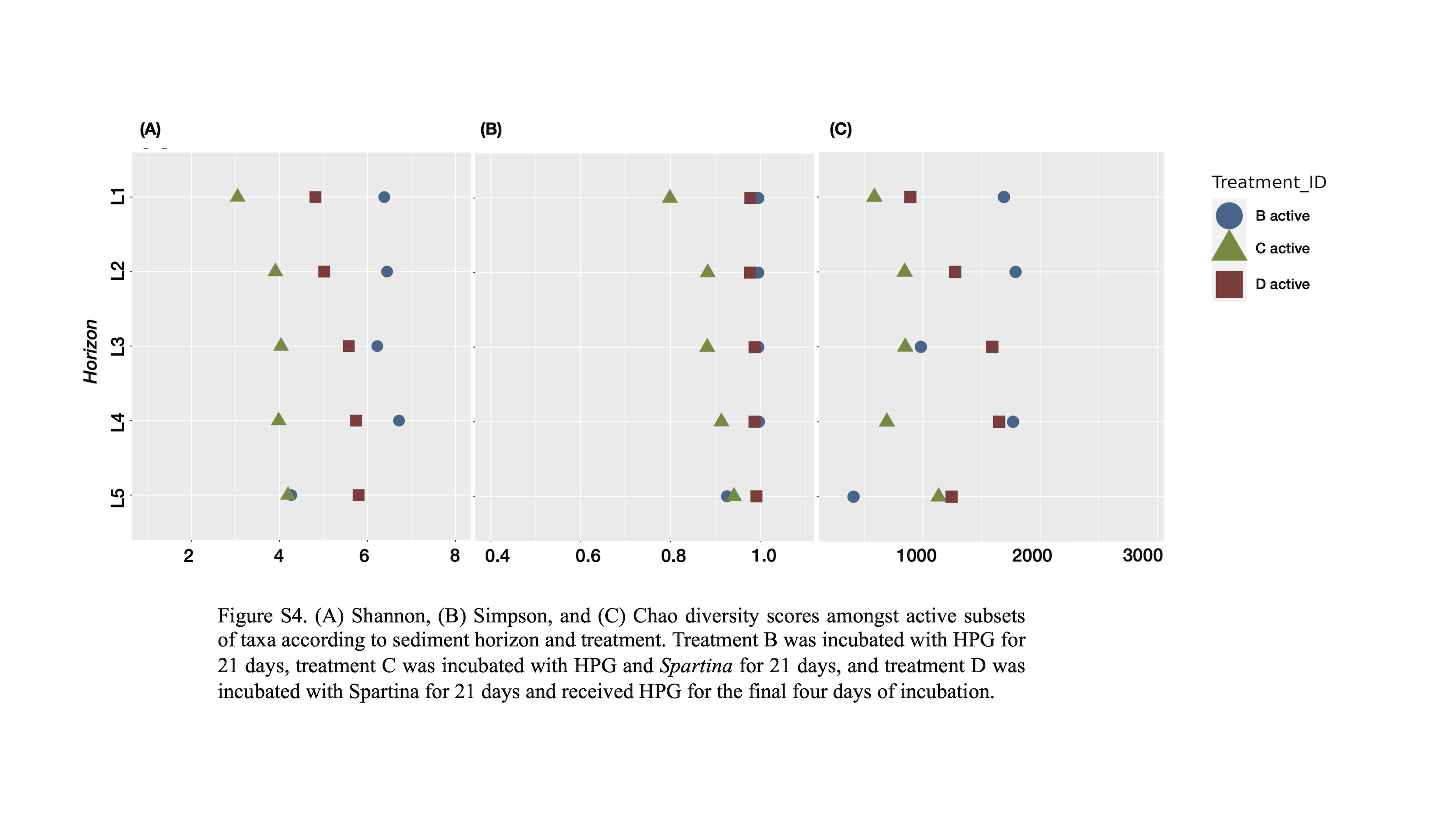

Supplement: Supplementary file 8 [file Image_4.TIFF]

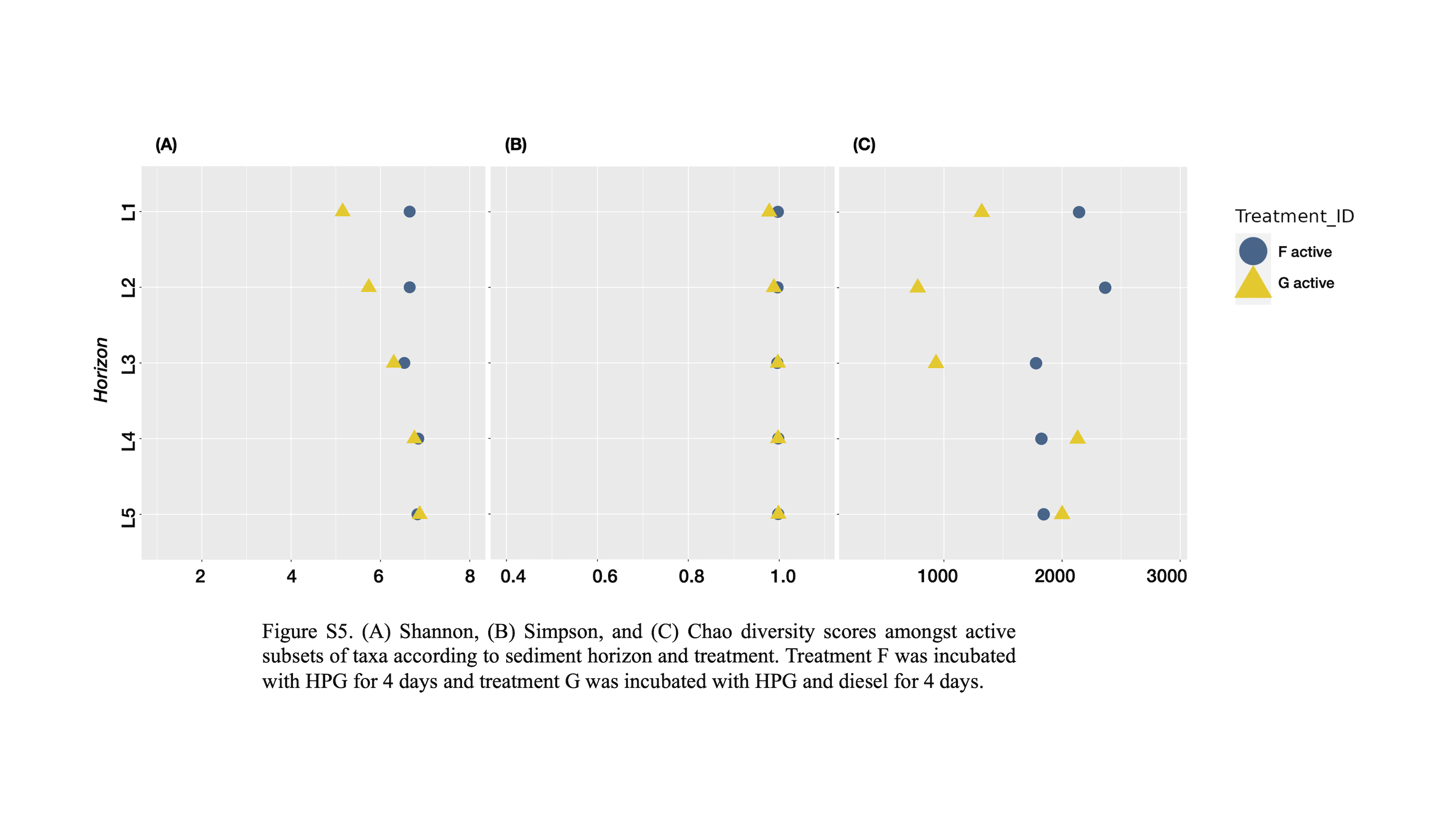

Supplement: Supplementary file 9 [file Image_5.TIFF]
